# Poultry feeds carry diverse microbial communities that influence chicken intestinal microbiota colonisation and maturation

**DOI:** 10.1186/s13568-020-01077-5

**Published:** 2020-08-15

**Authors:** Sarah Haberecht, Yadav S. Bajagai, Robert J. Moore, T. T. Hao Van, Dragana Stanley

**Affiliations:** 1 University of New England, Armidale, NSW 2351 Australia; 2 Institute for Future Farming Systems, Central Queensland University, Rockhampton, QLD 4702 Australia; 3 School of Science, RMIT University, Bundoora, VIC 3083 Australia; 4 Department of Microbiology, Monash University, Clayton, VIC 3800 Australia

**Keywords:** Feed, Microbiota, Colonisation, Chicken

## Abstract

Microbial colonisation of the gastrointestinal tract of newly hatched chicks starts at hatch, seeded from the immediate hatching environment, and quickly results in dense colonisation. The role of ecological factors in gut colonisation has been extensively investigated, as well as the role of micro- and macronutrients in supporting and selecting for bacterial species highly adapted for utilising those nutrients. However, the microbial community contained in poultry feed and its influence on colonisation and maturation of gut microbiota has not been directly addressed. In this study, we compared the microbiota found in poultry feed, with the microbiota of ileum, cecum and excreta, to identify substantial overlap in core microbiotas of the compared groups. We then investigated the microbiota present in raw feedstuffs: meat and bone meal, wheat, corn, canola, barley, soybean, millrun, sorghum, poultry oil, oats, limestone and bloodmeal from four geographically distinct feedstuff suppliers. Each of the feedstuffs had diverse microbial communities. The meat and bone meal and bloodmeal samples had the most complex and distinct microbial populations. There was substantial overlap in the phylogenetic composition found in the grain and seed samples: barley, canola, corn, millrun, oats, sorghum, soybean meal and wheat. Issues related to methodology, viability of microbial communities in the gut and feed, and the implications for biosecurity are discussed.
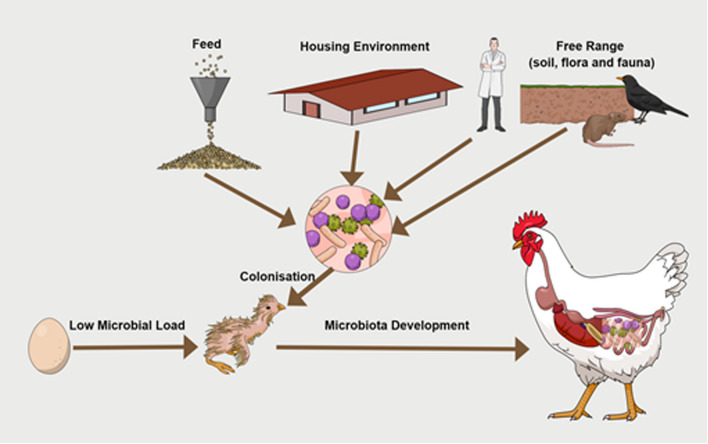

## Key points

- Rapid microbiota colonisation starts from birth, or hatch in poultry.
- Feed carries rich microbial community within and seeds the host during colonisation.
- In poultry feed ingredients, grains have similar microbiota.
- The meat and bone meal and bloodmeal had the most complex and distinct microbial populations..

## Introduction

Until recent advances in technology allowed us to sequence total DNA from any environmental sample and to identify almost all bacteria including uncultured, our knowledge was limited to a small proportion of bacteria we could grow and investigate using classic microbiology growth methods. Nowadays, instead of taking a fecal sample and pulling out only targeted bacteria on specially selected microbiological plates, we could see thousands of species in a sample and investigate their role in the gut. This revolutionised our knowledge of the intestinal microbiota and its role in health and digestion. We now know that the number of gut bacteria outnumber our own cells up to ten times and contribute around 99% of unique genetic material to our genetic pool (Cebra 1999; Fujimura et al. 2010; Joyce and Gahan 2014).

The poultry intestinal microbiota has evolved into its present form incorporating many different communities from the environment and the animals and humans they contact. This means that the phylogenetic composition of chicken gut microbiota strongly, but not entirely, overlaps with the microbiota of humans and other farmed animals. However, recent research into chicken gut microbiota has suggested that industrialization of chicken production has transformed the chicken gut microbiota to such an extent that modern commercial chicken microbiota is probably very different in composition to that which would be found in native jungle fowl, the wild precursors of the highly selected modern chicken, due to the un-natural hatching practices, with separation of chicks from hens and natural nest environments. In hatcheries, chicks are immediately exposed to bacteria different from bacterial communities to those that were selected in chicken guts and historically adapted to chicken as host (Stanley et al. 2013).

The practices of commercial poultry production expose newly hatched chicks to microbes from the hatchery environment, from human handlers, transport boxes, and transport vehicles, prior to arrival at the farm (Stanley et al. 2013). This process is typically carried out in the first days of life, during the period when there is a rapid increase in bacterial diversity and load in the gut. These environmental sources of bacteria appear to have a significant influence on the establishment of intestinal microbiota given that most significant colonisation in chickens occurs within the first few days post-hatch (Lu et al. 2003). In the absence of the natural chicken feeding, brooding and nesting habits (Crisol-Martinez et al. 2016) the chicken gut bacterial community becomes susceptible to the influences of human and environmental sources (Apajalahti et al. 2004; Stanley et al. 2013).

Until recently, it was believed that chicks are sterile *in ovo* and that colonisation begins post-hatch. The application of recent technological advances has suggested that, at least in some circumstances, there may be very low-level bacterial colonisation *in ovo* (Castaneda et al. 2019). There have been attempts to deliver probiotics *in ovo* (Abdel-Moneim et al. 2019; Wilson et al. 2019); however, bacterial load *in ovo* is very moderate and has production significance mainly in case of *in ovo* infection (Allan et al. 2018; Bradbury and Howell 1975). Bacterial colonization of the gut is likely to be a competitive process whereby the initial bacterial colonizers inhibit or promote the establishment of subsequent bacterial invaders by modifying the gut environment (e.g. pH) and/or cross-feeding metabolites that support or retard growth of other bacteria. The formation of the microbial community in chickens is very rapid with 10^8^ and 10^10^ bacteria per gram of contents in the ileum and the ceca respectively, one-day post-hatch. Numbers increase to 10^9^ and 10^11^ respectively by day three and remain high while continuously adapting and responding to environmental changes and host stressors (Baldwin et al. 2018; Lu et al. 2003; Stanley et al. 2013). This indicates that the first days post-hatch are critical for controlled and pathogen restricted microbial exposure. In this study, we investigated the potential role of microbiota from poultry feed in the establishment and development of chicken gut microbiota.

The food consumed by an animal has an important impact on the composition of gut microbiota; it supplies nutrients that the microbiota can use directly or which are derived from the host processing of the feed input (Fujimura et al. 2010). Digestible and simple carbohydrates in the gut are quickly absorbed and used by both host and microbiota in the small intestine, where they have the strongest influence on microbiota composition. However, dietary fibre, in the form of non-digestible carbohydrates (NDC), non-starch polysaccharides (NSP), resistant (non-digestible) oligosaccharides (RO) and resistant starch (RS), survive the passage through the small intestine largely unprocessed to reach the ceca and large intestine where they promote the growth of beneficial bacteria such as *Bifidobacterium* (Costabile et al. 2010; Holscher et al. 2015; Koecher et al. 2015; Lecerf et al. 2012; Whelan et al. 2005), *Lactobacillus* sp. (Costabile et al. 2010; Walton et al. 2010), *Akkermansia muciniphila* (Fruge et al. 2018) and *Faecalibacterium prausnitzii* (Benus et al. 2010; Roychowdhury et al. 2018). Some of the metabolic products derived from the bacterial digestion of dietary fibre have beneficial effects on intestinal and general health (Holscher et al. 2015; Koecher et al. 2015).

Unfortunately, unlike fibre-digesting bacteria, which are beneficial to the host, proteolytic intestinal bacteria in the chicken, such as *E. coli*, are often pathogenic (Tolckmitt 1957). King et al. (2009) identified *Enterococcus faecalis, Enterococcus gallinarum*, and *Proteus mirabilis* as frequently observed protease-secreting bacterial species in chicken. Bacterial metabolism of protein results in toxic metabolites. High protein content used in poultry diets may contribute to gut damage and is a predisposing factor in necrotic enteritis (Stanley et al. 2012, 2014). Herring et al. (2018) demonstrated that in humans high maternal dietary protein intake results in intrauterine growth reduction and embryonic death, due to the toxicity of ammonia, homocysteine, and H_2_S that are generated from amino acid catabolism. Recently, proteolytic species like *Bacillus subtilis* (Chen et al. 2009) have become popular protein digestion probiotics that aid proteolysis without pathogenic effects (Abdel-Moneim et al. 2020); however, the toxicity of the metabolites produced requires careful consideration when deciding on the protein content in poultry feed.

Most of the current knowledge on the role of microbiota in fat metabolism comes from human studies on the effects of high-fat diet and obesity. High-fat diets have not yet been extensively researched in poultry nutrition, from a microbiota perspective. High-fat diet consumption generally leads to an increase in *Firmicutes* and causes microbiota alterations clearly associated with obesity and intestinal diseases. A high-fat diet increases the number of fat-loving bacteria such as *Verrucomicrobia*, Deltaproteobacteria, *Ruminococcus*, *Lachnospiraceae*, and *Bacteroidaceae* (Hussain et al. 2019). Despite these bacterial groups being predominantly non-pathogenic or even beneficial to the host under normal diet circumstances, under high fat intake conditions cumulative metabolic products of these bacteria can result in multiple negative effects. High fat intake results in microbiota and host products that enhance gut permeability and result in chronic gut inflammation and predisposition to food allergy. This effect is mediated by fat-induced changes in the gut microbiota. Once a high fat diet increases fat-loving bacteria the host retains the community with increased ability to extract energy from food, as shown in human high fat diet and obesity studies (reviewed in Murphy et al. 2015).

Many studies have investigated the role of particular micro- and macro-nutrients on microbiota development in chickens and other animals, as well as the role of exogenous enzymes and other metabolites found in the feed. Other than the nutrient-driven influence of feed on microbiota development, the role of feed in contributing to the colonization of the gut with indigenous feed bacteria is underexplored. In this manuscript, we present evidence of the presence of diverse bacterial population in poultry feed rations that is continuously, from the first to the last day of life, seeding the poultry intestine with bacteria that are naturally present in feed and thus already adapted to digest and utilise components from that feed.

## Materials and methods

### Animal trial

The study was approved by the Animal Ethics Committee of Central Queensland University under the approval number A14/09-318. The animal trial used in this study was performed with a range of treatments with different probiotic supplementations. However, here we present a subsection of that data obtained from the 20 control birds and data from the feed that was provided to the birds from the hatch.

The birds were hatched in the Central Queensland University research facility hatchery using Ross Broiler 308 eggs provided by Bond Enterprises Hatchery, Toowoomba, Queensland. The eggs were hatched under relatively clean conditions and immediately moved to the poultry room where the feed was provided immediately post-hatch. The feed used was a Chicken Starter Diet (Red Hen, Laucke Mills, Australia) with no antimicrobials or coccidiostats and was used throughout the trial for 6 weeks. The feed was formulated to meet or exceed the National Research Council standards for broiler chickens (NRC 1994). All birds were fed ad libitum and had unrestricted access to drinking water. Birds were individually tagged by leg bands and weekly weights demonstrated weights equal or above the Ross 308 performance standards. Birds were euthanised at day 42 post-hatch (CO_2_, BOC, Australia) and dissected. Ileum and caecum contents were collected for microbiota analysis, flash-frozen in liquid nitrogen and stored at −  80  °C until further processing. Excreta samples were also collected by placing a specially made transparent divider into the pen without removing the bird from the pen. The clean paper towel was placed under the bird and excreta collected immediately after voiding. Forty-eight samples from 16 birds were successfully collected and sequenced for microbiota phylogenetic analysis.

### Feedstuff microbiota experiment

After confirming the presence of microbial communities in the feed used in the animal trial, we further investigated specific feedstuff components used in the poultry feed. The original poultry feed ingredients that were sampled included meat and bone meal, wheat, corn, canola, barley, soybean, millrun, sorghum, poultry oil, oats, limestone, bloodmeal, acid oil and tallow. Samples of each type of feed ingredient were sourced from four different suppliers from four distinct regions of Australia, three from the state of Victoria, which has a temperate climate, and one supplier from Queensland, a state with a warmer subtropical climate.

### DNA extraction

DNA was extracted from the feed, feed ingredients, ileal content, caecal content and excreta samples using the same method previously described (Bauer et al. 2019a, b). Briefly, 0.2 g of samples were lysed and purified using a DNA spin purification column (Enzymax LLC, Cat# EZC101, Kentucky, US). The DNA quality and quantity were estimated using a NanoDrop spectrophotometer.

### 16 s rRNA gene sequencing

The V3-V4 region of 16 s rRNA genes were amplified using ACTCCTACGGGAGGCAGCAG (forward) and GGACTACHVGGGTWTCTAAT (reverse) primers containing barcodes, spacers and Illumina sequencing linkers (Fadrosh et al. 2014). The sequencing library was prepared following the manufacturer’s protocol (Illumina Inc., San Diego, CA, USA). The 16 s rRNA amplicon sequencing was completed on the Illumina MiSeq platform using 2 × 300 bp paired-end sequencing.

The data was analysed using Quantitative Insights Into Microbial Ecology (QIIME v.1.9.1) (Caporaso et al. 2010). The Fastq-Join algorithm was used to combine the paired-end sequences, allowing no mismatches within the region of overlap. Phred quality threshold had a minimum score of 20. The Uclust (Edgar 2010) was used to pick the OTUs at 97% similarity and chimeric sequences were filtered using Pintail (Ashelford et al. 2005). Taxonomic assignments were performed against the GreenGenes database (v2013_8) using QIIME default parameters (DeSantis et al. 2006). The OTU abundance table was rarefied to calculate a UniFrac matrix. Calypso (Zakrzewski et al. 2017) was used for further downstream analysis and visualisation of the data using Hellinger transformed (Legendre and Gallagher 2001) OTU table.

The sequence data is publicly available at the MG-RAST database under a project ID mgp455839.

## Results

### Feed microbiota

The composition of microbiota in the feed was compared to the structure of the microbiota in the different gut sections in the birds fed the same feed. Feed microbial composition comprised of 4 phyla: *Actinobacteria* (47.6% of all reads), *Proteobacteria* (38.9% of reads), *Firmicutes* (11%) and *Bacteroidetes* (2.5%) spread across 50 genera including, in alphabetic order: *Arthrobacter, Acinetobacter, Aerococcus, Bacillus, Bifidobacterium, Blautia, Brachybacterium, Brevibacterium, Clostridium, Comamonas, Coprococcus, Corynebacterium, Dietzia, Enterobacter, Enterococcus, Facklamia, Frigoribacterium, Jeotgalicoccus, Lactobacillus, Lactococcus, Leuconostoc, Microbacterium, Oscillospira, Paenibacillus, Proteus, Pseudochrobactrum, Pseudomonas, Ruminococcus, Sphingobacterium, Sporosarcina, Staphylococcus, Streptococcus, Trichococcus, Turicibacter, Wautersiella* and 15 unknown genera.

**Fig. 1:**
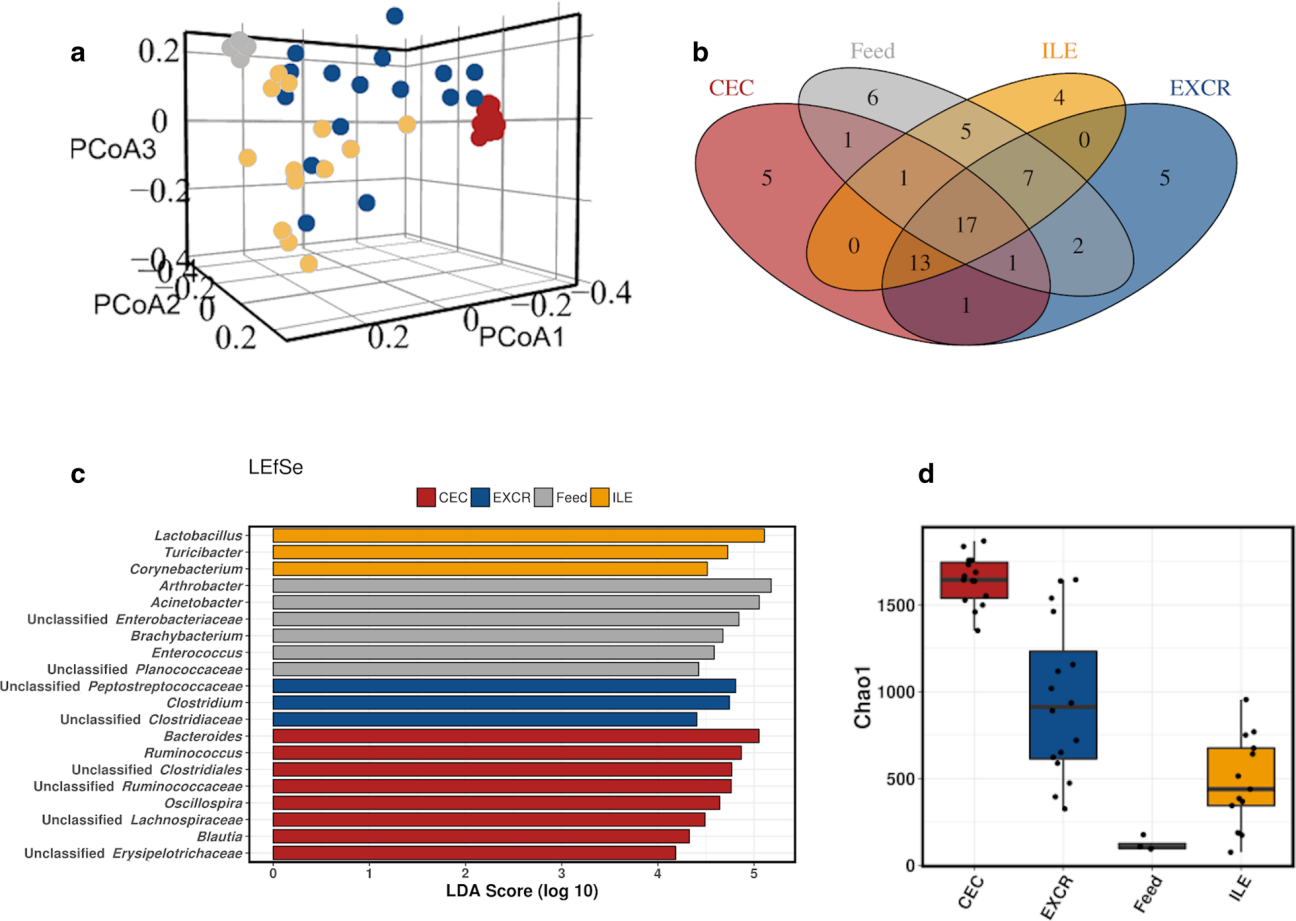
Comparison of feed microbiota with ileum, cecum and excreta microbiota. The Fig. presents 3D PCoA plot based on unweighted UniFrac distance (**a**); core microbiota at a genus level (**b**); LEfSe analysis of the top 50 genera (**c**) and Chao 1 microbial richness estimator (**d**). In all plots feed samples are represented in grey, cecum in red, ileum yellow and excreta in blue

The microbial composition of the feed was most similar to the microbiota of the ileum and excreta (Fig. 1a) and most distant from the cecal community. The core microbiota at genus level (Fig. 1b) showed the overlap in the genera present in the feed and both ileum and cecum, as well as with the excreta. The linear discriminant analysis (LDA) effect size method (LEfSe) was used to determine the genera most likely to explain differences between the feed and gut sections microbiota, via coupling standard tests for statistical significance with additional tests encoding biological consistency and effect relevance (Fig. 1c). The Chao 1 estimated richness of feed microbiota was very low compared to cecum and excreta samples (Fig. 1d), but comparable to some ileal samples.

### Individual feed ingredients carry distinctive microbial community

**Fig. 2:**
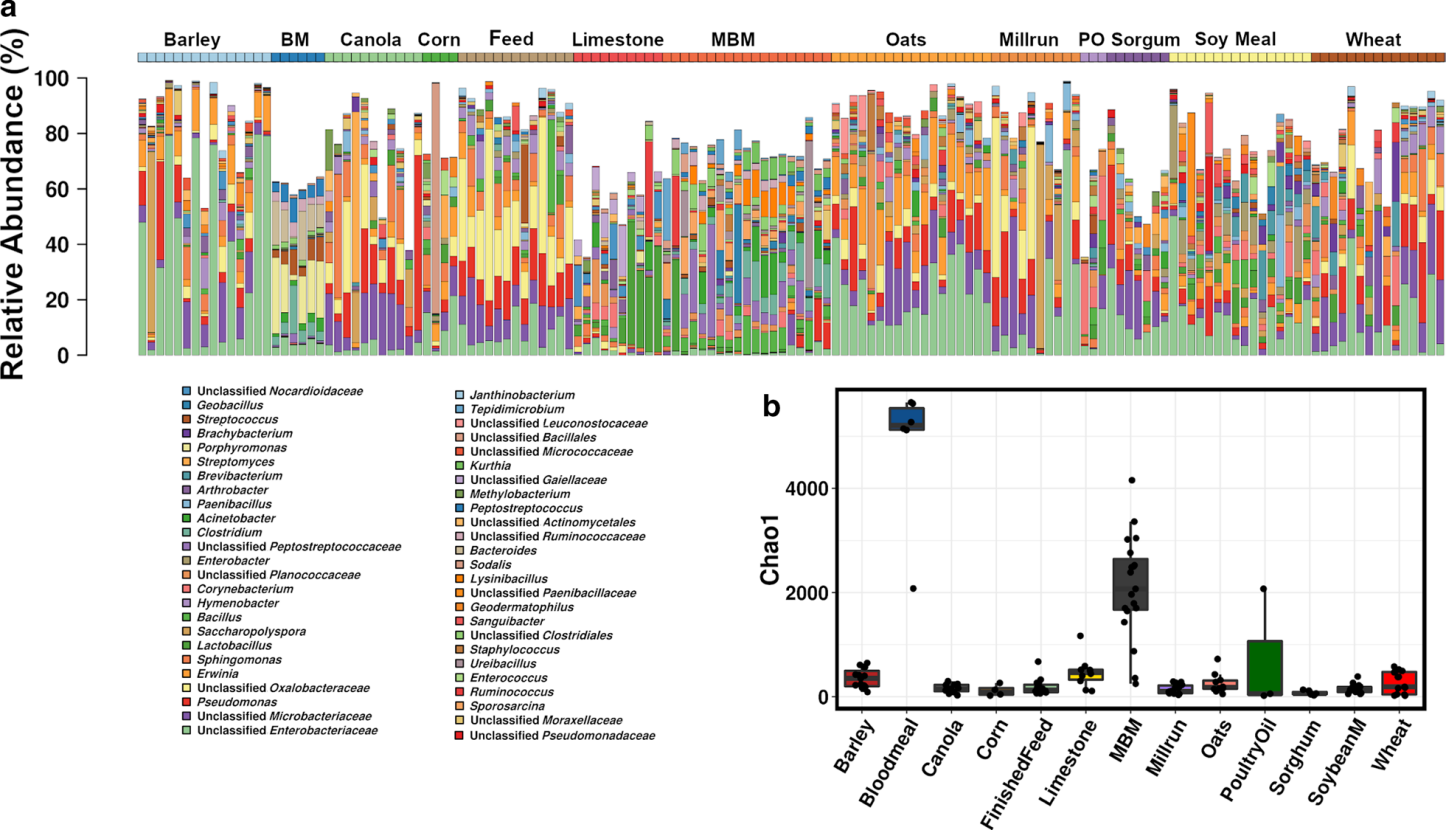
The feed ingredients microbiota composition and diversity. Genus level barchart shows the top 50 most abundant genera (**a**), and a Chao 1 richness estimator (**b**). *MBM * meat and bone meal, *BM* blood meal, *PO* poultry oil, ***Feed*** finished mixed feed

To determine the potential origins of the microbiota found in the whole formulated feed ration the microbiota composition of the component ingredients of the feed were analysed. Independent-samples of each of the ingredients were sourced from four different locations in Australia (Fig. 2). Figure 2a and b, show clear differences in genus level composition of feed ingredients, as well as in their estimated richness with bloodmeal and meat and bone meal showing more complex microbial richness compared to other feed ingredient groups.

**Fig. 3:**
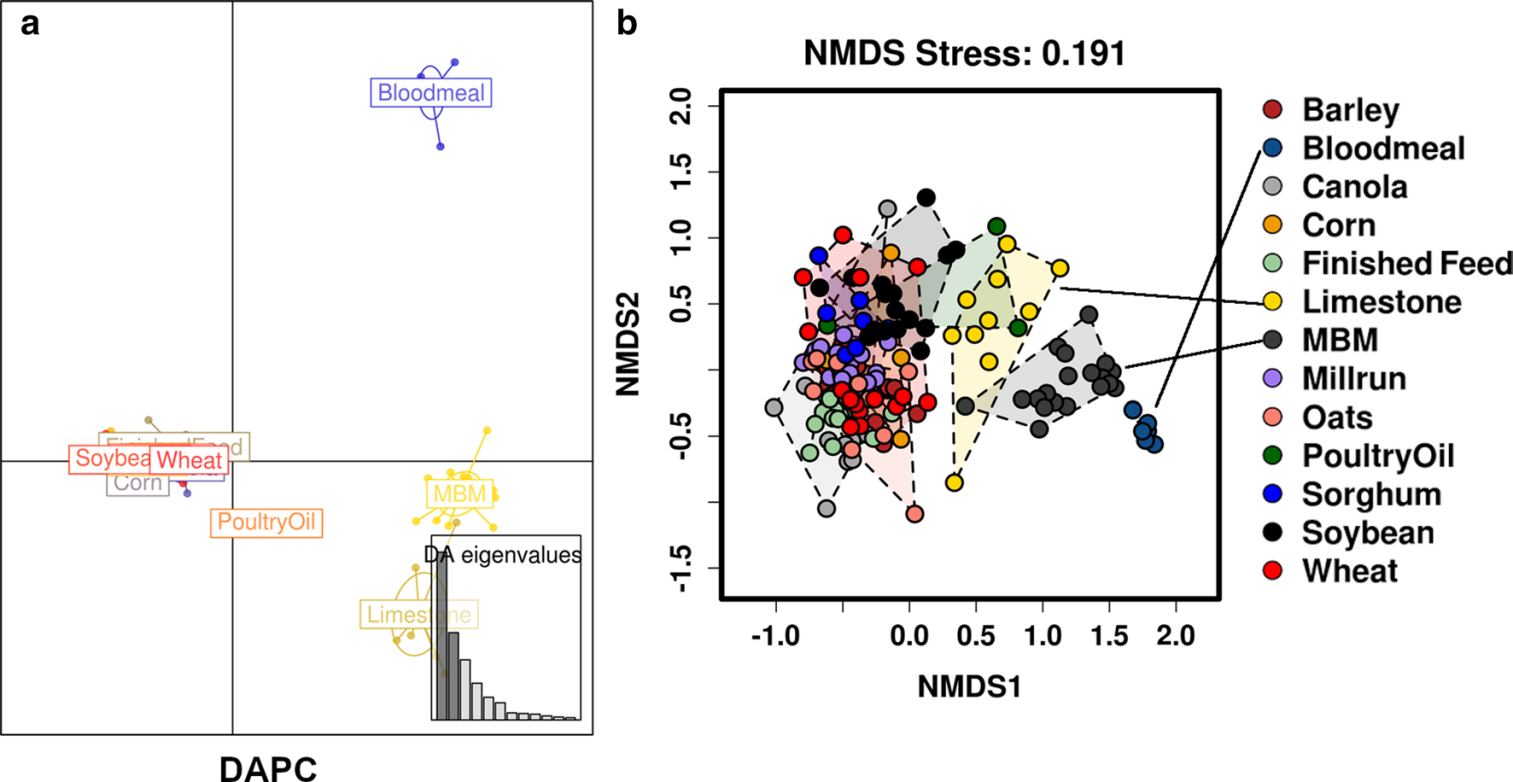
Multivariate presentation of feed ingredients microbiota similarities, via DAPC (**a**) and NMDS (**b**) plots

The similarities and differences in microbiota compositions were further investigated by Discriminant Analysis of Principal Components (DAPC) and Non-Metric Multidimensional Scaling (NMDS) multivariate analysis (Fig. 3a,b). This showed that bloodmeal, meat and bone meal, and limestone, had the most distinct microbial communities, followed by poultry oil, whereas barley, canola, corn, millrun, oats, sorghum, soybean meal and wheat clustered together into almost entirely overlapping groups.

**Fig. 4:**
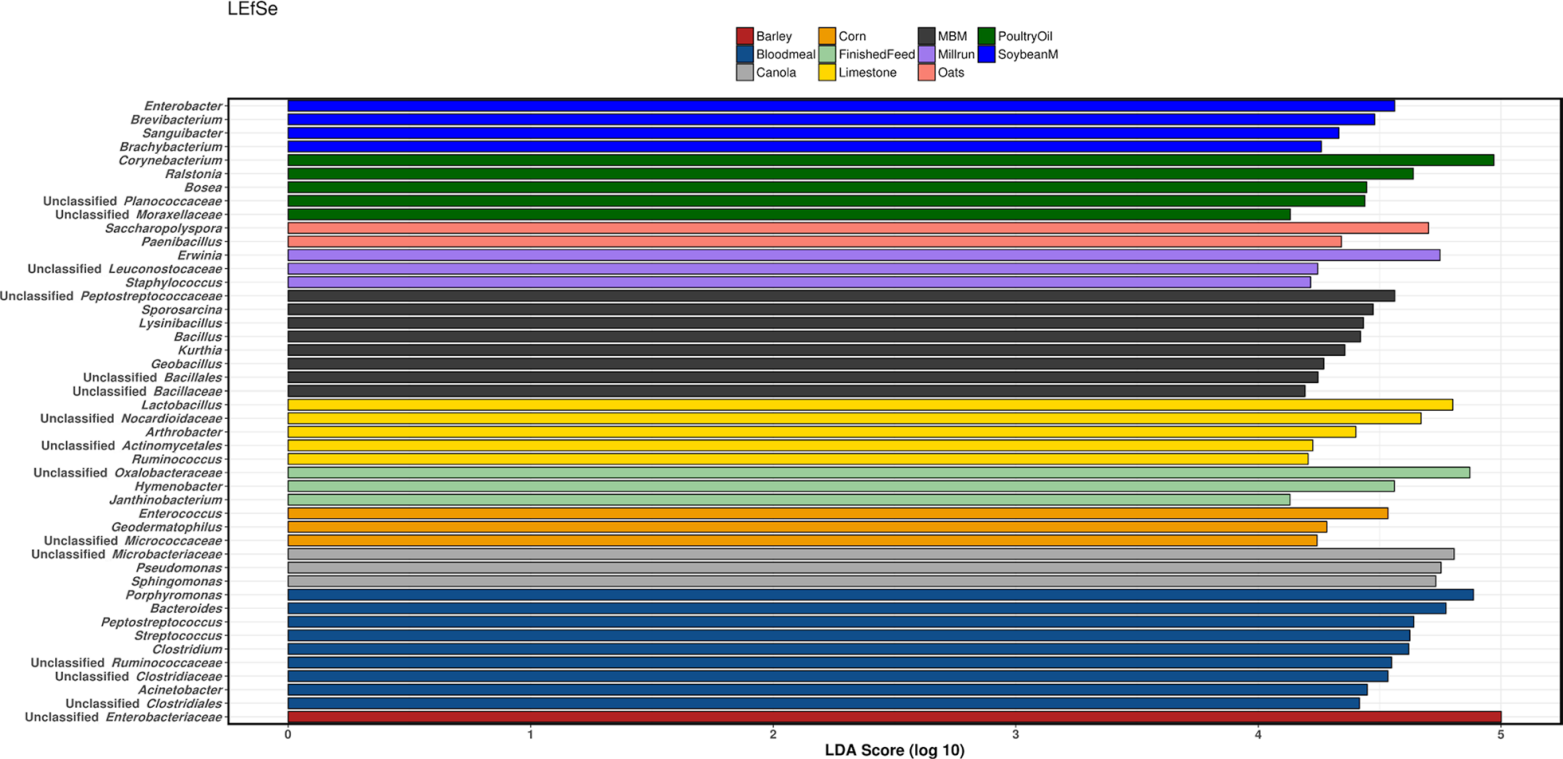
Linear discriminant analysis effect size method (LEfSe) of the top 50 genera in feedstuffs

Based on LEfSe the genera most likely to explain differences between each type of feed ingredient indicate that some groups of pathogens populating the bird GIT may originate from specific feedstuff, with *Clostridium* and *Streptococcus* identified as representatives of bloodmeal (Fig. 4).

**Fig. 5:**
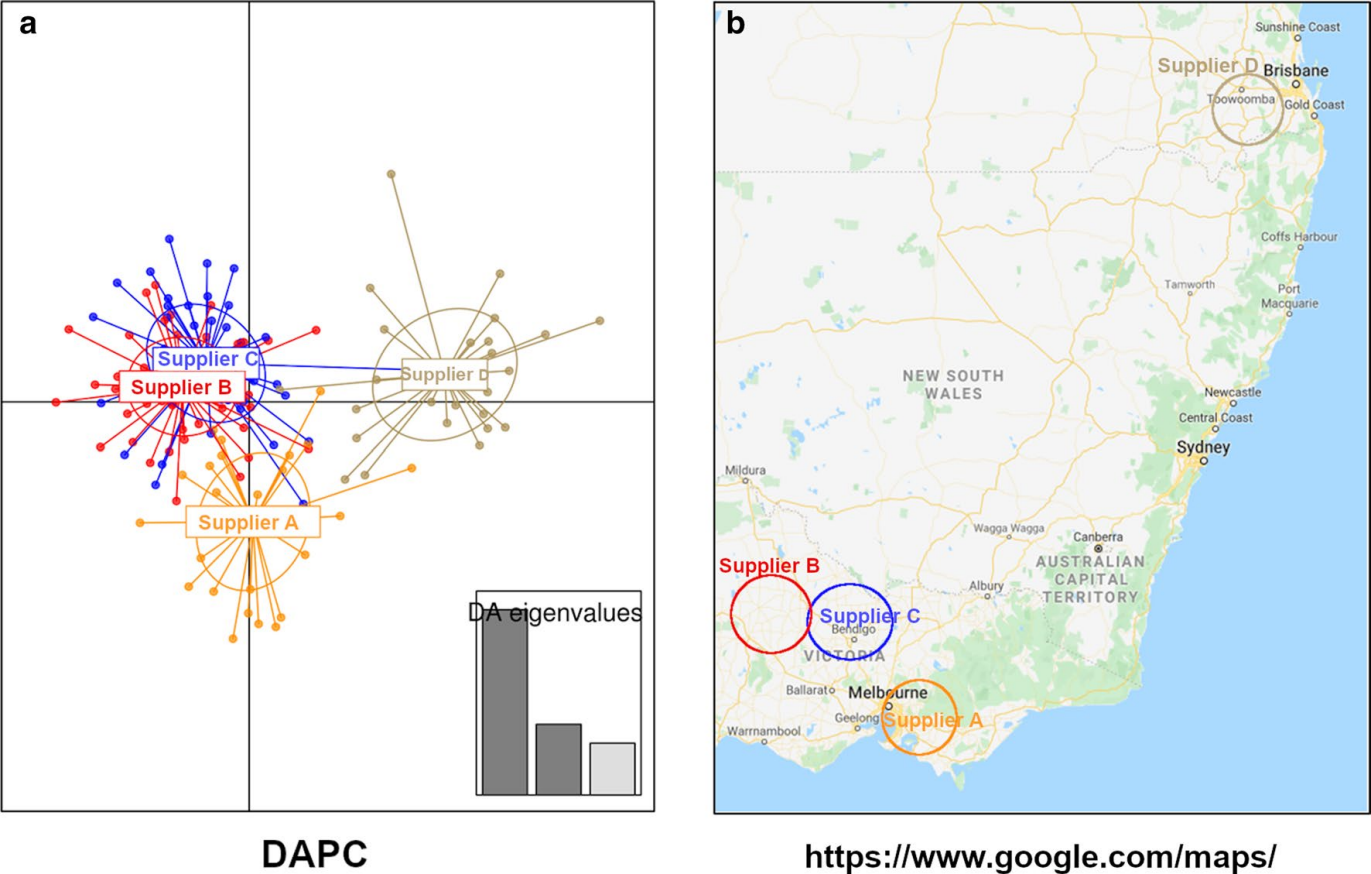
Discriminant Analysis of Principal Components (DAPC) of feedstuffs from the different suppliers (**a**) and a GoogleMap (**b**) view of their geographical region

The influence of the feed ingredients supplier environmental differences and/or climate conditions on the microbiota in feedstuffs is presented in a DAPC plot (Fig. 5). Surprisingly, the differences in feedstuffs microbiota presented in the DAPC plot resemble the geographical position of suppliers and may be influenced by the differences between the processing facilities but also climate and geographic region from which the ingredients have been grown and produced.

## Discussion

It is now well established that an appropriate, healthy, microbiota provides individuals with numerous nutritional benefits, intestinal mucosa development, pathogen protection and immune system maturation (Stecher and Hardt 2008; Young 2012; Zhu et al. 2002). Although the mainstream knowledge on gut microbiota came from human research, it is understood that intestinal bacterial inhabitants of chickens play both similar and poultry-specific, influential roles (Stecher and Hardt 2008; Young 2012; Zhu et al. 2002). Although, in some cases, there may be small populations of bacteria in the gut of embryos *in ovo* the main events of the establishment of the chicken intestinal microbiota community starts immediately post-hatch. Based on the current literature on poultry gut colonisation, the environment, rather than parental influence plays the major role in chicken gut maturation (Stanley et al. 2013). The microbiota in chickens is considered fully formed within the first weeks of life but it continues to mature and to respond to stressors and environmental challenges throughout the life of birds.

**Fig. 6:**
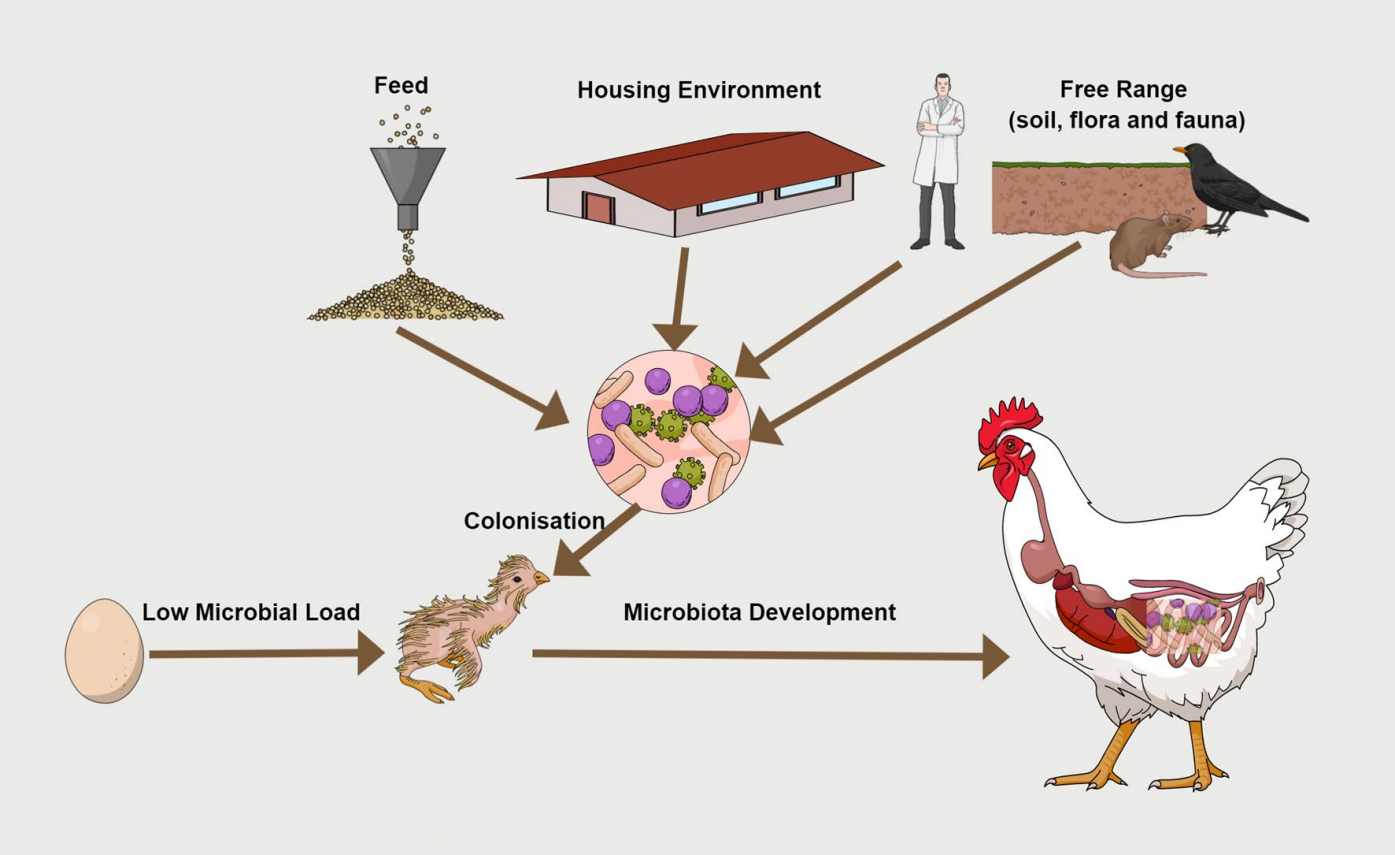
Feed contains a diverse microbial community and is an active bacterial coloniser in poultry

The type of the production system defines the environment and type of feed that birds are exposed to and hence has a major influence on the microbiota, especially in free-range systems that replace strict biosecurity with exposure to the soil, grass and other plant microbiota, but most concerning, microbiota of rodents, wild birds and other animals, and increases pathogen load in birds; all presenting challenges for free-range production (Biasato et al. 2018; Islam et al. 2019; Ocejo et al. 2019). Until now the feed has been usually discussed in poultry gut maturation as a growth medium that will support a nutrient determined cohort of intestinal microbes. This study now brings a new dimension to the role of feed in microbiota formation, as a source of colonizing bacteria (Fig. 6).

The microbial community contained in poultry feed may play an important role in the colonisation of poultry gut microbiota as the study found a large overlap in the core microbiota of the feed and the birds’ ileum, caecum and excreta. Our results also indicate that there is a substantial overlap in the microbial composition of grains and seeds: barley, canola, corn, millrun, oats, sorghum, soybean meal and wheat almost entirely overlap; while blood meal, meat and bone meal and limestone stand quite apart with more distinct microbiotas. Each batch of feed ingredient differs in the composition of the microbiota it carries and hence each batch of formulated feed will also differ in microbiota composition. Some of the ingredients carry bacteria which are potentially pathogenic. These findings suggest that it would be prudent to consider and monitor feed microbial community, particularly in starter diets used in the period when the core microbiota is formed and stabilised.

It is worth noting how poorly researched the field of microbial colonisation of feed ingredients used in poultry and human feed is. A Google Scholar search on keywords “grain” and “microbiota” (January 2020) returned only 4 manuscripts, all investigating the role of grains consumed in the diet on microbiota structure. Similar outputs are found when using specific grains. On the other hand, the feed can be easily contaminated with microbes as feed spoilage is not uncommon out in field situations. While biosecurity measures are implemented to variable degrees, feed ingredients in milling environments are not always well protected from birds, rodents, insects and other potential microbial exposures. It was believed that the process of pelletising will remove most of the bacteria. However, in addition to the fact that brief heating of feed during pelletising cannot remove microbial spores, the pelletising process is far from sterilisation and will remove most, but not all, viable bacteria. Its antimicrobial efficiency will be varying between the different processing systems. It is also notable that feed can be easily colonized, post-pelletising, from external and pelletising surviving microbiota. This will strongly depend on the packaging, humidity, temperature and other finished feed storage conditions.

Another question that needs to be addressed is how much of the microbial signal identified by 16 s rRNA gene analysis represents viable bacteria. DNA from the dead bacteria would be subjected to natural degradation but still, depending on time frames and storage conditions, significant amounts of amplifiable DNA from dead bacteria could be present. Of course, this can also be an issue with microbiota analysis of gut samples, although in that case it is well established that DNA is efficiently digested and degraded by gastric juice and pepsin (Liu et al. 2015). We were able to grow bacteria on rich brain-heart infusion media from all of the feed samples we investigated, however, culturing is strongly restricted by the media and anaerobic/aerobic environment used. Therefore, it would certainly not be readily possible to identify culturable bacteria of all the genera identified in feed ingredients. Although there are other methods capable of determining bacterial viability via sequencing and vital staining methodologies (Young et al. 2017) they are not commonly in use and face different challenges and methodological issues.

This study shows that further research into feed as a source of random, or as the means of targeted colonisation of poultry gut, could be productive. Investigating feed microbiota will bring new challenges, unlike the diverse and dense microbial populations present in intestinal samples, many of the feed ingredients are hard to process for DNA isolation and for some only small amounts of bacterial DNA can be recovered. Amplicon and whole metagenome sequencing methods are limited in resolution and cannot reliably detect bacteria that make up only a small fraction of the microbiota. Resolution depends on sequencing depth but in most studies, a presence at less than 0.01% is unlikely to be reliably detected. Therefore, for some significant bacteria, for example, pathogens such as *Clostridium perfringens* or *Salmonella*, it may be necessary to use more sensitive methods, such as culturing or specific PCR, to detect in feed ingredients. Control of viable pathogens in feed should be considered as a standard part of production system biosecurity.

## Data Availability

Sequencing data is publically available on MG-RAST Metagenomics Analysis Server Database (https://www.mg-rast.org/) with full sample annotation under project ID mgp455839.
